# Tobacco-specific nitrosamine 1-(N-methyl-N-nitrosamino)-1-(3-pyridinyl)-4-butanal (NNA) causes DNA damage and impaired replication/transcription in human lung cells

**DOI:** 10.1371/journal.pone.0267839

**Published:** 2022-05-16

**Authors:** Altaf H. Sarker, Bo Hang

**Affiliations:** Biological Systems and Engineering Division, Lawrence Berkeley National Laboratory, Berkeley, CA, United States of America; University of Connecticut, UNITED STATES

## Abstract

Thirdhand smoke (THS) is a newly described health hazard composed of toxicants, mutagens and carcinogens, including nicotine-derived tobacco specific nitrosamines (TSNAs), one of which is 1-(N-methyl-N-nitrosamino)-1-(3-pyridinyl)-4-butanal (NNA). Although TSNAs are generally potent carcinogens, the risk of NNA, which is specific to THS, is poorly understood. We recently reported that THS exposure-induced adverse impact on DNA replication and transcription with implications in the development of cancer and other diseases. Here, we investigated the role of NNA in THS exposure-induced harmful effects on fundamental cellular processes. We exposed cultured human lung epithelial BEAS-2B cells to NNA. The formation of DNA base damages was assessed by Long Amplicon QPCR (LA-QPCR); DNA double-strand breaks (DSBs) and NNA effects on replication and transcription by immunofluorescence (IF); and genomic instability by micronuclei (MN) formation. We found increased accumulation of oxidative DNA damage and DSBs as well as activation of DNA damage response pathway, after exposure of cells to NNA. Impaired S phase progression was also evident. Consistent with these results, we found increased MN formation, a marker of genomic instability, in NNA-exposed cells. Furthermore, ongoing RNA synthesis was significantly reduced by NNA exposure, however, RNA synthesis resumed fully after a 24h recovery period only in wild-type cells but not in those deficient in transcription-coupled nucleotide excision repair (TC-NER). Importantly, these cellular effects are common with the THS-exposure induced effects. Our findings suggest that NNA in THS could be a contributing factor for THS exposure-induced adverse health effect.

## Introduction

Recently, a new type of smoke exposure called thirdhand smoke (THS) gained significant public attention and interest. THS is defined as the residual tobacco smoke pollutants in second hand smoke (SHS) that remain attached on surfaces and dust after active smoking [1]. Nicotine, the most abundant smoke component, deposits on indoor surfaces during smoking and reacts with pollutants in the indoor environment to form a mix containing toxic and carcinogenic chemicals [1,2]. More specifically, surface-bound nicotine reacts with nitrous acid (HONO), a common atmospheric species that is emitted from indoor combustion appliances and smoking, to produce tobacco-specific nitrosamines (TSNAs) including NNA (4-(Methylnitrosamino)-4-(3-pyridyl)butanal), NNK (4-(methylnitrosamino)-1-(3-pyridyl)-1-butanone) and NNN (N’-nitrosonornicotine) [1,2]. Substantial levels of TSNAs are detected on surfaces inside smoker’s home, vehicles, skins, cloths and these levels often increase over time with frequent smoking [1–4]. Some of these compounds such as NNK and NNN are potent carcinogens in animal studies [5], however, the risk of NNA, mainly detected in THS and rarely found in mainstream smoke (MSS) or second-hand smoke (SHS), remains largely unknown. NNA may be adsorbed through the skin by detection in urine of two predicted metabolites 4-(methylnitrosamino)-4-(3-pyridyl)-1-butanol (*iso*-NNAL) and 4-(methylnitrosamino)-4-(3-pyridyl)butyric acid (*iso*-NNAC) when NNA was applied to the skin in mouse studies [6]. It is reasonable to suppose that such exposure could take place in humans through dermal contact, ingestion or inhalation and that children and infants are the most at risk due to their metabolisms and age-specific behavior putting them in close proximity to carpet and furniture [1].

Although considerable progress has been made in THS research, the adverse biological and health effect caused by THS exposure is still poorly understood. We previously reported the formation of DNA strand breaks in THS-exposed human cell lines by the alkaline Comet assay [7] and γ-H2AX immunofluorescence [8]. Consistent with these results, we observed that THS exposure increased micronuclei (MN) formation [9], a marker of genomic instability. These cellular effects are consistent with THS exposure induced adverse effects *in vivo* [10]. Furthermore, THS exposure also induced replication and transcription stress that are linked with an elevated risk of cancer and premature aging, respectively [9].

THS constitutes a mixture of toxicants, mutagens and carcinogens, most of which are also present in MSS and SHS. However, a component specifically enriched in THS but absent in SHS may represent THS specific features and can be used for risk assessment. To identify such component in THS, we characterized NNA, a THS specific nitrosamine. Its mutagenic potential using 6-thioguanine assay [11] and induction of strand-breaks with Comet assay [7] has been reported. Together with NNK and NNN, NNA has also been tested in A/J mice for tumorigenicity, but it showed low activity that was attributed to its highly reactive aldehyde group leading to its inability to reach cellular targets [12].

To better understand the genotoxicity of NNA, we investigated the formation of oxidative DNA damage and DSBs in lung epithelial BEAS-2B cells exposed to NNA at different doses. We also investigated the consequences of cellular replication and transcription from exposure of lung cells to NNA. We chose lung epithelial BEAS-2B cells since the lungs would be a major target site for NNA-containing THS and we could compare the effect of NNA with our recently reported similar studies using whole THS samples [9]. Finally, we also examined the role of nucleotide excision repair (NER), a major DNA repair pathway involved in repair of bulky DNA lesions, in the defense of NNA-derived cellular effects. The data obtained further support the formation of DNA adducts upon NNA exposure. From the results reported here, we conclude that NNA is genotoxic at appropriate dose range, and may play a role in THS exposure-induced adverse effects on health.

## Methods

### Human cell lines, antibodies and reagents used in this study

Transformed non-tumorigenic human bronchial epithelial cells (BEAS-2B) were from ATCC (Manassas, Virginia). Antibodies used were 53BP1 (A300-272A, Bethyl Laboratories), phospho-RPA32 S4/S8 (A300-245A, Bethyl Laboratories), pATM S1981 (ab81292, Abcam), anti-HIF-1α (A300-286A, Bethyl Laboratories) and rabbit anti-XPA (sc853, Santa Cruz). NNA (1-(N-methyl-N-nitrosamino)-1-(3-pyridinyl]-4-butanal) were procured from Toronto Research Chemicals (Cat# M325650, Ontario, Canada) and dissolved in methanol.

### Cell extract preparation and western blotting

BEAS-2B cells were cultured in DMEM containing 10% FBS (Invitrogen), 2 mM L-Glutamine (Gibco) and 1% penicillin/streptomycin (Cellgro) and maintained at 37°C with 5% CO_2_. For induction of DNA damage, cells were typically grown about 75% confluency, washed twice, and incubated with different doses of NNA (0.1, 1 and 10 μM) in serum-free DMEM. For Western analysis of specific proteins, cells were trypsinized, washed with PBS and lysed in a buffer containing 100 mM Hepes-KOH (pH 7.5), 250 mM KCl, 5 mM MgCl_2_, 1 mM EDTA, 0.5% Igepal (v/v), 10% glycerol and a protease inhibitor cocktail (Roche). The DNA and RNA in the suspensions were digested with 5 units/ml benzonase (Novagene) for 30 min on ice and appropriate amount of SDS-loading dye added. The samples were heated at 94°C for 5 min and separated by either 4–8% or 4–12% SDS-acrylamide gels (Invitrogen) depending on the size of proteins interested. Proteins were transferred to a nitrocellulose membrane and probed with the indicated antibodies. Immunoblots were quantified by Versadoc 4000MP and Quantity One software (BioRad).

### Long amplicon (LA)-QPCR assay

After exposure to varying doses of NNA (0.1, 1 and 10 μM) or DMEM only (control) for 24 h, the cells were harvested for genomic DNA extraction using the Qiagen Genomic-tip 20/G kit (Qiagen) per the manufacturer’s instructions. This kit is able to minimize DNA oxidation during the isolation step and has been used previously for LA-QPCR assay-based studies [7,13]. After quantification, equal amounts of genomic DNA were digested with two *E*. *coli* base excision repair (BER) enzymes, Fpg and EndoIII (Trevigen) which are able to remove a variety of oxidized purine and pyrimidine bases and induce strand breaks by cleaving the phosphodiester bond with their associated AP lyase activity [7,14].

To examine the formation of oxidative DNA damage induced by NNA, we amplified two genes, DNA polymerase β (*POLB*) and hypoxanthine phosphoribosyl transferase 1 (*HPRT*), and a non-coding fragment (NCF) from chromosome 4, by LA-QPCR with genomic DNA from NNA-exposed human cells as described previously [7,14]. LongAmp Taq DNA polymerase (NEB) was used to amplify a 9.4 kb fragment of *POLB*, a 10.4 kb fragment of *HPRT* gene, and 9.6 kb NCF from chromosome 4, respectively. The LA-QPCR reactions were performed from the same stock of genomic DNA to avoid variations of PCR amplifications. A typical amplification reaction contains 50 ng of Fpg/EndoIII digested genomic DNA template. The final PCR reaction conditions were at 94°C for 30s (94°C for 30s, 58°C for 30s, 65°C for 10 min) for 25 cycles to amplify the non-coding sequence and 30 cycles for *POLB* and *HPRT* amplification; and final extension at 65°C for 10 min. Since amplification of a small region would be relatively independent of oxidative DNA damage (low probability of the formation of the lesions), a small DNA fragment for *non-coding region* (294 bp), *POLB* (192 bp) and *HPRT* (250 bp) was also amplified for normalization of the data obtained with the large fragments. All the primers for amplification of the large and short fragments were standardized by Van Houten’s group (14) and listed in “Table 1”. The amplified products were resolved and visualized using agarose gel electrophoresis and quantitated with Varsadoc system (Bio-Rad). The data were plotted as histograms with relative amplification as Y-axis with mock-exposure control arbitrarily set to 100.

**Table 1:** Primers used in this study.

Primers	Amplification	Nucleotide sequence 5’- 3’	Purpose
NCF_L- F	Noncoding	CACAGCTGAGGCCCGTTGGG	LA-QPCR
NCF_L-R	Noncoding	CCGGGCCTGTGGGTTAGGGA	LA-QPCR
NCF_S-F	Noncoding	CAGCTATCCCAGCACCATTTA	Short-PCR
NCF_S-R	Noncoding	GTATGGCAACCTGCTGATAGT	Short-PCR
POLBL-F	*POLB*	CGTTCTGGGATACCCT	LA-QPCR
POLBL-R	*POLB*	CTGGAGTAGGGCCAAGAA	LA-QPCR
POLBS-F	*POLB*	AGTGGGCTGGATGTAACCTG	Short-PCR
POLBS- R	*POLB*	CCAGTAGATGTGCTGCCAGA	Short-PCR
HPRL-F	*HPRT1*	TGGGATTACACGTGTGAACCA ACC	LA-QPCR
HPRL-R	*HPRT1*	GCTCTACCCTCTCCTCTACCGTC	LA-QPCR
HPRS-F	*HPRT1*	TGCTCG AGATGT GAT GAA GG	Short-PCR
HPRS-R	*HPRT1*	CTG CAT TGT TTT GCC AGT GT	Short-PCR

### Indirect immunofluorescence

For measuring DSBs, immunofluorescence analysis was carried out as described previously [9,13]. For 53BP1 immunostaining, cells grown in 4-well chamber slides (Nunc Lab-Tek) were exposed to NNA (1 and 10 μM) or mock exposure (DMEM only) for 24 h and then washed three times with PBS. Cells were fixed with 4% para-formaldehyde (PFA) and 0.3% Triton-X-100 in PBS prior to permeabilization with PBS containing 0.5% Triton X-100. Cells were blocked with 2% bovine serum albumin (BSA) and incubated overnight at 4°C with rabbit anti-53BP1 (A300-272A, Bethyl Laboratories) antibody. Following washes, samples were incubated with secondary antibodies conjugated to Alexa Fluor 488 (Molecular Probes) and with 4′,6-diamidino-2-phenylindole (DAPI) to stain the nuclear DNA. Slides were mounted in Vectashield, and images were captured using a Zeiss LSM 710 confocal microscope (Carl Zeiss Inc.). Images within the same data set were captured with the same exposure time, so that the intensities were within the linear range and could be compared between samples.

### DNA synthesis assay

DNA synthesis as measured by incorporation of 5-ethynyl-2’-deoxyuridine (EdU) was essentially the same as previously described [9,15]. The *Click-iT EdU cell proliferation assay* (Invitrogen) was employed for detection of cells undergoing DNA synthesis. Cells were plated (30,000 cells/well) in a 4-well chamber slide and grown overnight followed by exposure to NNA (1 and 10 μM) in serum free media or mock exposure (only DMEM) for 24 h. DNA synthesis levels were determined following incubation with 10 μM EdU for 4 h [9,15] by direct addition to the serum-free medium. EdU incorporation was visualized using highly specific *ClickIT* reaction with Alexa Fluor 488 dye according to the manufacturer’s protocol (Invitrogen, Cat #c10337). Briefly, cells were fixed with 3.7% PFA for 15 min followed by permeabilization with 0.5% triton X-100 for another 20 min at room temp. After extensive washing with PBS, cells were blocked with 2% BSA for 1 h at room temp followed by incubation for 30 min protected from light with reaction cocktail containing Alexa Fluor 488-azide, per the *Click-iT* EdU imaging protocol (Invitrogen). Slides were washed, and samples were counterstained with DAPI for nuclear staining to a final concentration of 1 μg/ml. Images were captured using a Zeiss LSM 710 confocal microscope and analyzed with ImageJ (NIH) software. In each experiment, >100 cells per condition were analyzed to determine percentage of cells with the fluorescent signal.

### Micronuclei (MN) assay

The cytokinesis-blocked micronuclei assay was used to investigate formation of micronuclei (MN) in bi-nucleated cells [16,17]. BEAS-2B cells were grown in a 6-well plate and exposed to 1 and 10 μM NNA or mock exposure for 48 h. Cytochalasin B was added immediately after NNA and mock-exposed cells at a concentration of 3 μg/ml, followed by further incubation for 48 h. Cells were trypsinized, centrifuged and re-suspended in 7 ml of 0.075 M potassium chloride for 10 min followed by fixation with 3 ml of 100% methanol for 1 h at room temp. The cells were centrifuged and again fixed twice with acetic acid/methanol (1:3). Fixed cells were dropped onto wet slides and stained with Diff Quick following the protocol as described [9,17]. For each sample, micronuclei formation in 100 bi-nucleated cells was scored and plotted. Sample identity was blinded prior to scoring.

### Small Interfering RNA (siRNA) knock-down assay

siRNA sequence and transfection methods are the same as described previously [9,18]. Briefly, XPA siRNA (5’-GCTACTGGAGGCATGGCTA-3’) and non-specific control siRNA (5’-GATTCGAACGTGTCACGTCAA-3’) were purchased (Qiagen) and 40 nM were transiently transfected into BEAS-2B cells (~70% confluent) in a 6-well plate with lipofectamine^TM^ RNAiMAX (Invitrogen) on two consecutive days. Cells were re-plated and incubation for 72h and tested for XPA knock-down by Western. XPA depleted BEAS-2B cells were exposed to 10 μM NNA for 24h and processed for IF analysis.

### RNA synthesis assay

BEAS-2B cells were grown in a 4-well chamber slide and exposed to NNA (1 and 10 μM), UV (20 J/m^2^) or mock exposure (DMEM only) for 24 h. RNA synthesis levels were determined by directly adding of 1 mM 5-ethynyl uridine (EU) to the culture medium followed by incubation for 2 h. Incorporation of EU was visualized by *Click IT* conjugation of Alexa Fluor 488 according to the manufacturer’s protocol (Invitrogen Cat# c10329) and as described earlier [9,15]. Images were obtained and quantified as described [9]. For each experiment, >20 randomly selected cells from 40x magnification were used for analysis.

### Recovery of RNA synthesis

Both repair proficient BEAS-2B and NER deficient (XPA knock-down**)** cells were grown in a 4-well chamber slide and exposed to NNA (10 μM) or mock exposure for 24 h. At the end of exposure both mock and NNA-exposed samples from BEAS-2B and siXPA down-regulated BEAS-2B cells were proceed for EU labeling as described above. For recovery of RNA synthesis assay both BEAS-2B and siXPA down-regulated cells were washed with PBS after 24 h exposure to 10 μM NNA were allowed to repair for another 24 h with complete DMEM (10% FBS) medium followed by EU incorporation and visualization by *Click IT* conjugation of Alexa Fluor 488 according to the manufacturer’s protocol (Invitrogen, Cat# c10329) and the method in [9]. Images were obtained and quantified by Image J as described above. For each experiment, >25 randomly selected cells from 40x magnification were used for analysis.

### Statistical analysis

Mean value and SD or SEM error bars are shown. Unpaired, two-tailed t-test was used to determine statistical significance between groups. Significance was defined as a p value less than or equal 0.05. All analyses were performed using GraphPad PRIZM (GraphPad, CA) software.

## Results

### NNA exposure caused accumulation of oxidative DNA damage in cultured human lung cells

**Fig 1:**
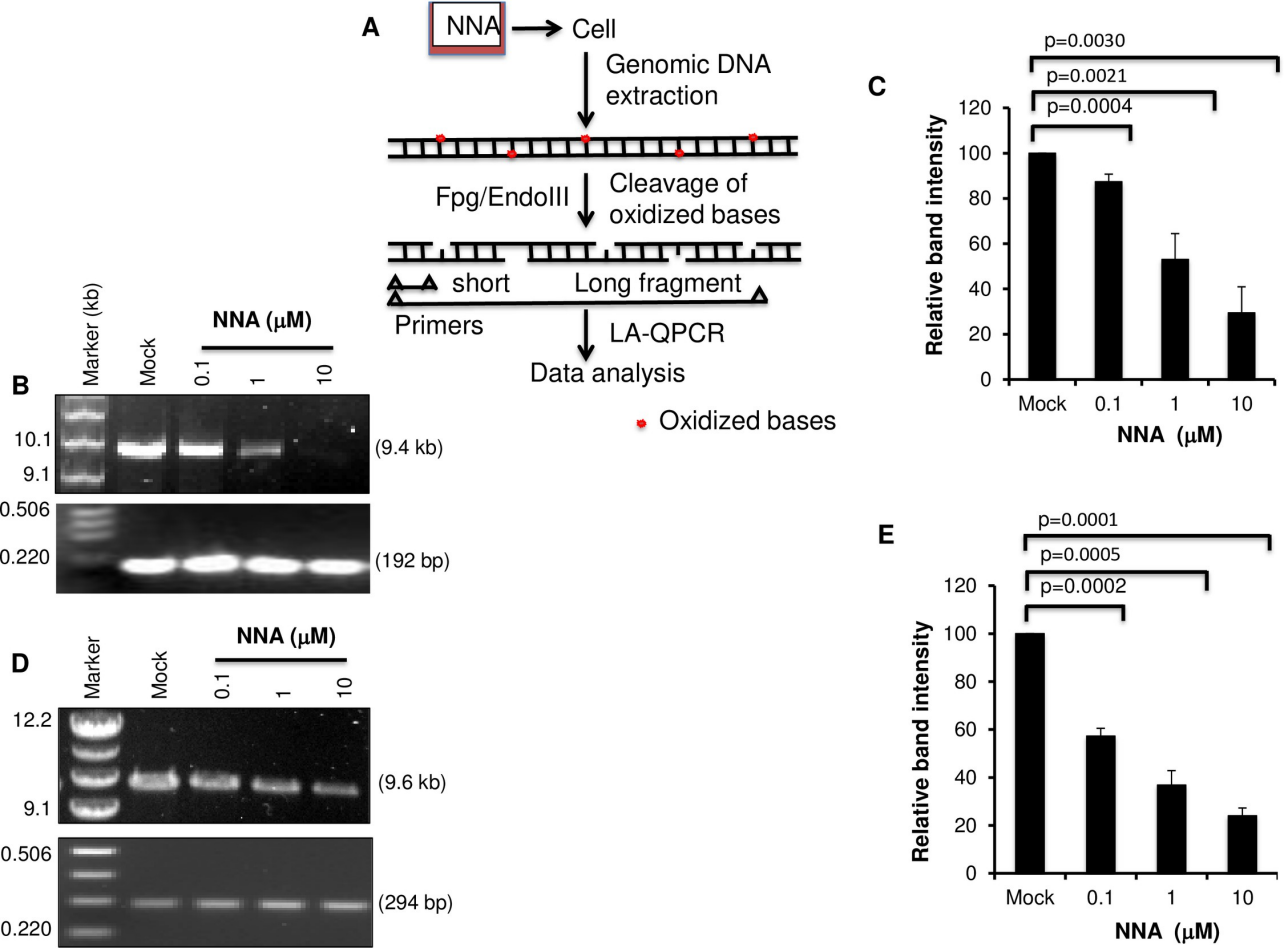
NNA exposure induced oxidative DNA damage as measured by LA-QPCR. BEAS-2B cells were exposed to three concentrations (0.1, 1 and 10 μM) of NNA for 24 h as indicated at the top, followed by processing of the genomic DNA with Fpg and EndoIII as described in the Methods. Mock exposure (DMEM only) was performed in parallel. (A) Schematic representation of NNA exposure induced oxidative DNA damage analysis. (B) Amplification of the long (9.4 kb) and short (192 bp) amplicon of *POLB* gene (Chromosome 8; Accession AF491812). (C) Amplification of the large fragment was normalized to the corresponding short fragment. The bar graph represents the normalized data as relative band intensity with mock-exposed sample arbitrarily set to 100. (D) Amplification of the long (9.6 kb) and short (294 bp) amplicon of a noncoding fragment (NCF) from chromosome 4 (Accession AC023886.7). (E) Amplification of the large fragment was normalized to the corresponding short fragment. The bar graph represents the normalized data as relative band intensity with mock-exposed sample arbitrarily set to 100. Each experiment repeated three times and one representative gel figure is shown. Error bars represent ±SEM of the mean.

Formation of pre-mutagenic oxidative lesions 8-oxo-dG from *in vitro* reaction of NNA with deoxyguanosine (dG) led us to test whether NNA is also capable to induce similar lesions in the cell [1]. We exposed cultured lung epithelial BEAS-2B cells to 0.1, 1.0 or 10 μM NNA for 24 h. These doses and the time point were selected based on our previous data showing that such dose range induced DNA strand breaks in the Comet assay 24 h after NNA exposure [7]. Genomic DNA was initially isolated from the exposed cells followed by treatment with *E*. *Coli* Fpg/EndoIII before analysis of the damage by LA-QPCR to excise oxidized bases and to generate single-strand breaks, thereby preventing PCR amplification by the polymerase. A dose-dependent decrease in the amplification of both 9.4 kb *POLB (*Fig 1A & 1B) and 9.6 kb NCF (Fig 1C & 1D) fragments was observed, indicating increasing levels of oxidative DNA damage in both the coding and non-coding fragments upon NNA exposure. We also found significantly decreased amplification of 10.4 kb *HPRT* fragment (S1 Fig), another gene previously used to measure DNA damage [7,13]. The decrease in percentage was between 70–75% for the highest concentration (10 μM) tested. In each cases amplification of the short fragments were nearly unchanged independent of exposure doses because the probability of damage in a much shorter fragment is greatly lower than in a longer fragment and was used for normalization.

We also examined whether the NNA doses used above would increase HIF-1α level, a marker of reactive oxygen species (ROS) production [13,19]. BEAS-2B cells were exposed to 1 and 10 μM NNA for 24 h followed by Western analysis with an antibody recognizing HIF-1α (A300-286A, Bethyl Laboratories). As shown in S2 Fig, both doses efficiently induced 2-fold increase in HIF-1α level. However, control TFIIH subunit XPB remains unchanged, suggesting selective upregulation of HIF-1α reflecting an increased ROS production after NNA exposure [13,19]. Taken together, these results indicate that NNA exposure causes a significant increase in oxidative DNA damage in human lung BEAS-2B cells.

### NNA exposure induces DSBs in cultured human lung cells

**Fig 2:**
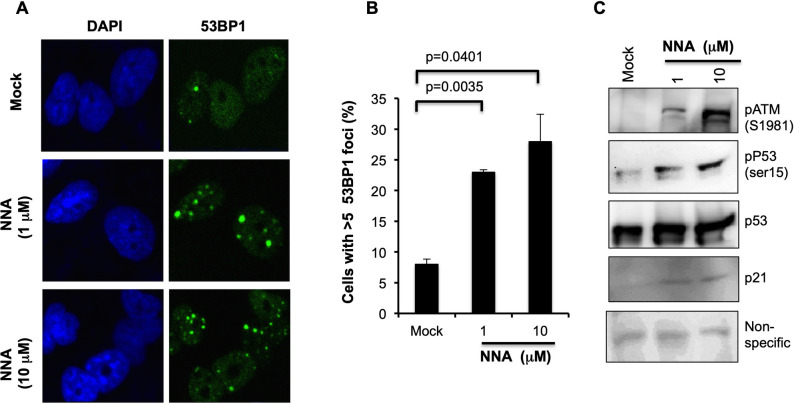
NNA exposure induced DSBs and activation of DSB response machineries. (A) Representative images of 53BP1 foci staining 24 h after two doses of NNA (1 and 10 μM) exposure. Mock exposure control performed in parallel and represented at the top panel. (B) Quantification of 53BP1 foci 24 h after NNA exposure. Number of cells with more than five foci were counted and plotted as percent. Significance test was performed with two-tailed t test in comparison to mock exposed cells (p value <0.05). (C) Activation of ATM and its downstream p53 signaling pathway following exposure of BEAS-2B cells to the indicated doses of NNA for 24 h was analyzed by Western with anti-pATM (Ser1981) antibody. The same sample was probed for phosphorylation of p53 using anti-pp53 (Ser15), p53 and p21 antibodies. A non-specific band from the same gel was used as loading control.

We previously reported increased DNA strand breaks in human HepG2 cells exposed to THS or NNA using the alkaline Comet assay [7]. It should be noted that the strand breaks detected by this assay include both single- (SSBs) and double-strand breaks (DSBs). To confirm if NNA induces DSBs, we exposed BEAS-2B cells with 1 and 10 μM NNA for 24 h and measured 53BP1 foci, a marker of DSB, by immunofluorescence (IF). We found a dose-dependent increase in the number of cells with more than 5 foci compared to the mock exposure control (Fig 2A). Quantification of the cells with more than 5 foci indicated a 2.4-fold (p = 0.0035) and 3.5-fold (p = 0.0401) increase over control following exposure to 1 and 10 μM NNA, respectively (Fig 2B). To the best of our knowledge, this is the first demonstration that NNA exposure induces the formation of DSBs, the most lethal DNA lesions leading to genomic instability and cancer.

### Activation of p53 pathway by NNA exposure

Induction of DSBs upon NNA exposure prompted us to test if p53 pathway is activated. Increased DSBs suggested the activation of ATM, a master regulator of DNA damage responses. Upon DNA damage, ATM autophosphorylates its S1981 residue leading to its dissociation from an inactive dimer to the active ATM monomer, which then phosphorylates downstream proteins involved in DNA repair, apoptosis and cell cycle checkpoints [20]. To examine ATM activation by NNA exposure, BEAS-2B cells were exposed to 1μM and 10 μM NNA or mock exposure (DMEM) for 24 h and ATM phosphorylation was monitored by Western analysis using phospho-specific antibody (ab81292, Abcam). Activation of ATM was detected at both 1 and 10 μM NNA doses tested (Fig 2C, panel 1). One of the key substrates for ATM activation is the phosphorylation of p53, a protein regulating cell cycle check point and apoptosis in response to damage. To test if p53 is activated we examined p53 phosphorylation in 1 and 10 μM NNA-exposed BEAS-2B extracts by Western blot. We found increased phosphorylation of p53 at Ser15 with both doses tested (Fig 2C, panel 2) but the total p53 did not changed significantly. In addition, the protein level of the p53 transcriptional target p21 was also increased (Fig 2C, panel 4). A non-specific band from the same membrane was used as protein loading control.

### NNA exposure causes replication stress in human lung cells

**Fig 3:**
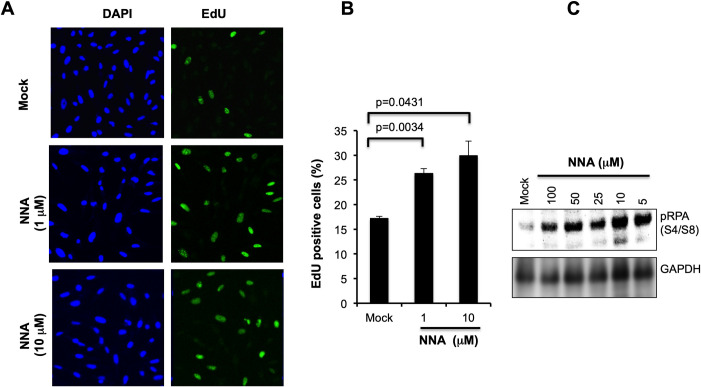
Proliferation analysis following exposure of BEAS-2B cells to NNA. (A) Replication stress was monitored by Western analysis with RPA32 phosphorylation using phospho-RPA32 (S4/S8) antibody. GAPDH was used as a loading control for normalization of pRPA32 signal. (B) Cells were exposed to 1 and 10 μM NNA for 24 h followed by EdU incorporation for 4 h, fixed, permeabilized, detected with Alexa Fluor 488 by the *Click-iT* reaction (Invitrogen) and imaged. DAP1 stain confirmed total cell counts. Mock (DMEM only) exposure performed in parallel served as control and represented at the top panel. (C) Quantification shown as percent EdU positive cells. Data are presented as the mean ± SD from N = 3.

NNA induction of DSBs as shown above (Fig 2) and of bulky base lesions *in vitro* [1,21], both of which are potent blocks to replicating polymerases, prompted us to test for increased replication stress in human lung cells exposed to NNA. We examined the status of RPA32 phosphorylation, which is induced during replication stress. RPA32 is phosphorylated at S4/S8 residue, and phospho-RPA32(S4/S8) is required for replication checkpoint arrest [22]. As shown in Fig 3A, markedly increased phospho-RPA32(S4/S8) following exposure of BEAS-2B cells to a range of NNA doses for 24 h was consistent with a high degree of replication stress in these cells induced by NNA exposure.

If the cells are undergoing replication stress as a consequence of NNA exposure, then the fraction of cells undergoing DNA synthesis would also be affected. DNA replication was detected by incorporation of the thymidine analog EdU as measured by IF following *Click* chemistry [13,15]. BEAS-2B cells were exposed to 1 and 10 μM NNA for 24 h followed by a 4 h pulse with 10 μM EdU as described earlier [13]. In mock exposed cells we observed only 17% EdU positive cells. In contrast, 1 μM and 10 μM NNA-exposed samples we observed 26% (1.53-fold; p = 0.0034) and 30% (1.8-fold; p = 0.0431) EdU positive cells, respectively (Fig 3B & 3C). These data support that a significant fraction of replicating BEAS-2B cells exposed to NNA were not able to exit from S-phase.

### Induction of micronuclei following exposure of lung cells to NNA

**Fig 4:**
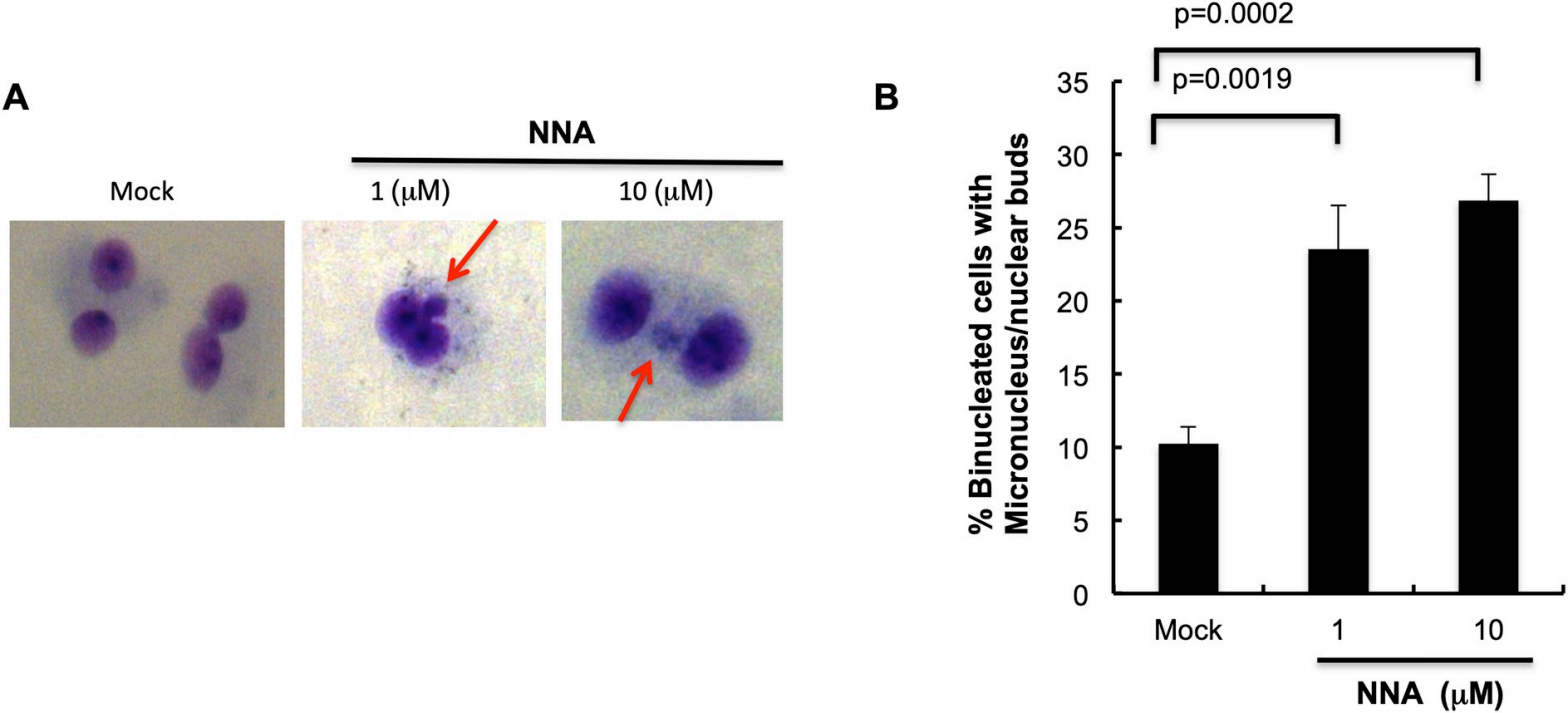
Genomic instability was induced by NNA exposure. (A) BEAS-2B cells were exposed to the indicated doses of NNA or mock exposure for 48 h and micronuclei formation was measured in each population. Microphotographs of bi-nucleated (Cytochalasin B-treated) cells stained with Diff Quick are shown. Mock (DMEM only) exposed cells were used as control. (B) Quantification of the micronuclei in bi-nucleated cells and plotted. Data represent the mean of ±SD for N = 2. Significance was obtained with student t test in comparison to mock exposed control (p<0.05).

NNA exposure-induced DSBs and replication stress suggest a possible increase in micronuclei (MN), an indicator of genomic instability and cancer risk [17]. BEAS-2B cells were exposed to 1 μM and 10 μM NNA for 48 h followed by addition of Cytochalasin B to block cytokinesis, then further incubation for another 48 h in the presence of NNA, and then scoring for MN in bi-nucleated cells [16,17]. Significantly increased MN formation was observed following exposure of BEAS-2B cells to both 1μM (2.3-fold; p = 0.0019) and 10 μM (2.6-fold; p = 0.0002) NNA doses compared to the mock exposed control (Fig 4A & 4B). This effect strongly suggests that NNA is a potent inducer of genomic instability.

### NNA exposure impairs transcription

**Fig 5:**
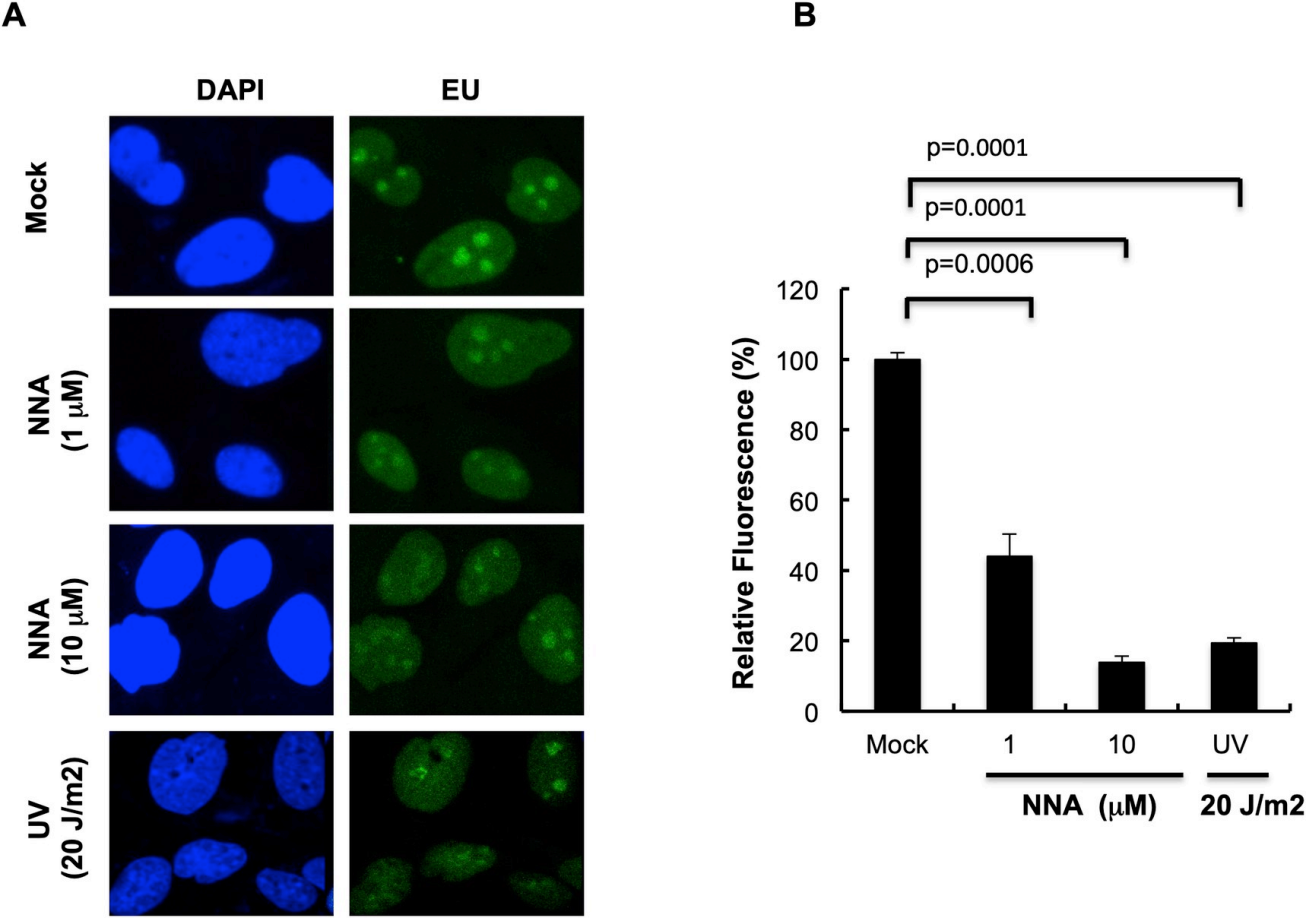
Basal transcription impaired following exposure to NNA. (A) BEAS-2B cells were exposed to NNA for 24 h with the indicated doses or mock treated, and ongoing RNA synthesis was measured by EU incorporation and *Click IT* reaction. As a control for transcription inhibition, cells were irradiated with 20 J/m^2^ UV and EU was added 1 h after exposure. Representative images obtained at 40x magnifications are shown. (B) Quantification by ImageJ of the EU fluorescence intensity of 20 randomly picked cells from mock, NNA exposed or UV irradiated samples. Data represent the mean of ± SEM for N = 3.

Bulky DNA lesions such as those produced by UV radiation are potent blocks to transcription by stalling elongating RNAPII (RNAPIIo), thus affecting RNA synthesis. TC-NER is the mechanism to repair the bulky lesions for efficient recovery of RNA synthesis and cell survival [22–24]. Because of the potential of NNA to form bulky adducts in DNA [1,21], we tested whether NNA exposure affects RNA synthesis by measuring the incorporation of EU. Significantly reduced RNA synthesis as measured by fluorescence intensity was observed after 24 h of exposure to either 1μM or 10 μM NNA (Fig 5A & 5B). Quantification showed 56% and 87% reduction of the fluorescence intensity in 1 μM and 10 μM NNA-exposed samples, respectively. A similar effect was also observed in the same type of cells receiving a high dose (20 J/m^2^) of UV radiation [known to produce bulky DNA adducts] when analyzed 1 h post-UV incubation (Fig 5A & 5B). These results suggest that bulky DNA lesions generated in lung cells following exposure to NNA strongly block elongating RNAPIIo and thus reducing ongoing transcription of active genes.

### RNA synthesis recovery following NNA exposure depends on intact NER

**Fig 6:**
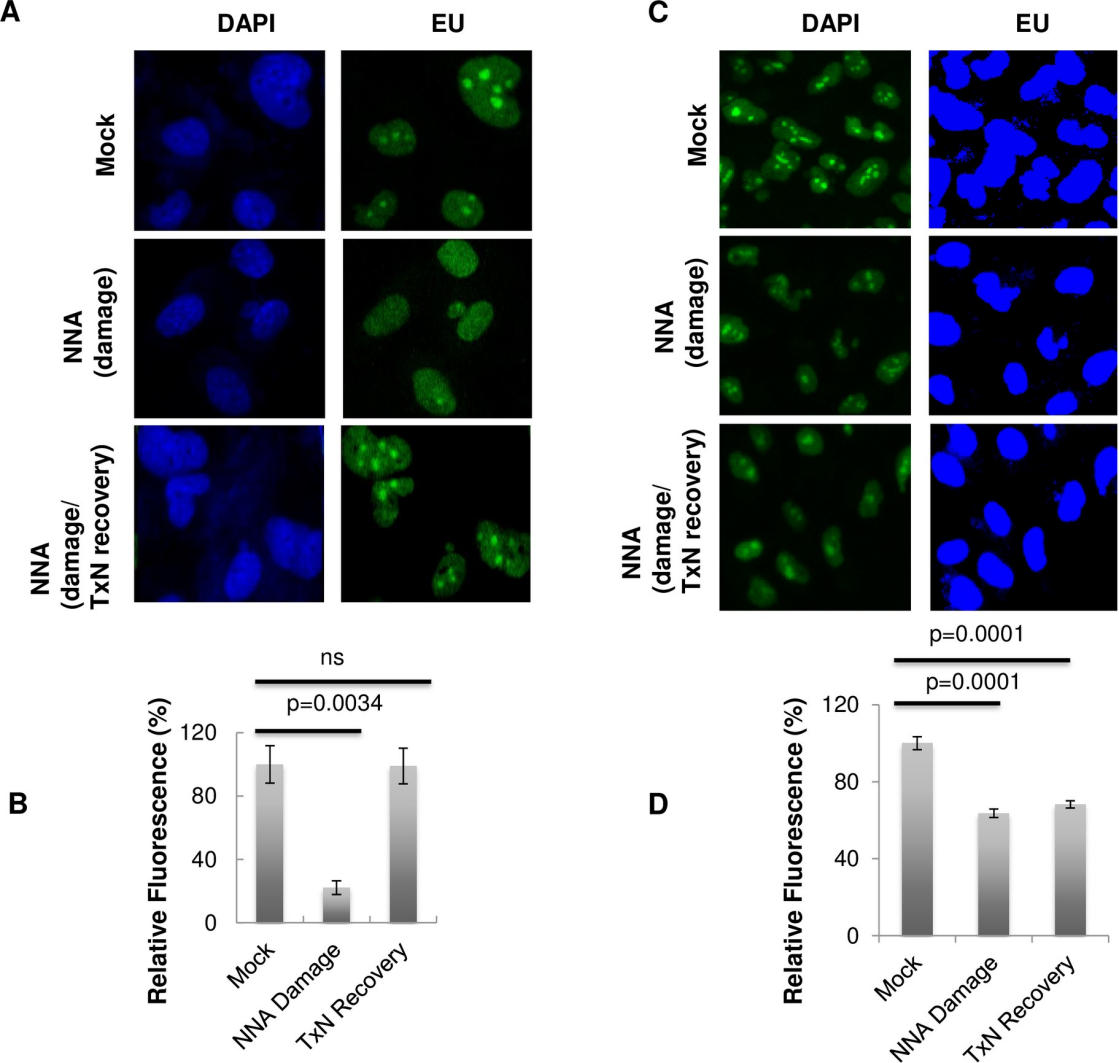
Impaired basal transcription by NNA exposure recovered in normal but only slightly recovered in NER defective cells. (A) BEAS-2B cells were exposed to 10 μM NNA (Panel 2 &3) or mock exposure (Panel 1) for 24h followed by EU incorporation (in mock and panel 2) in the presence of NNA and processed for *ClickIT* reaction. For recovery of RNA synthesis (Panel 3 at the left), after exposure to 10 μM NNA for 24 h, cells were washed with PBS and allowed to repair for another 24 h with DMEM containing 10% FBS followed by EU incorporation and imaging. (B) Quantification by ImageJ of the EU fluorescence intensity of >25 randomly picked cells from mock, NNA exposed or recovered samples. Data represent the mean of ± SEM for N = 3. (C) XPA was down-regulated from BEAS-2B cells by siRNA were exposed to 10 μM NNA for 24 h followed by EU incorporation and imaged as in (A). Recovery of RNA synthesis (Panel 3 at the right), after exposure to 10 μM NNA for 24 h cells were washed with PBS and allowed to repair for another 24 h with complete DMEM medium followed by EU incorporation and imaged. 40x magnification images are shown. (D) Quantification using ImageJ of the average fluorescence intensity of >20 cells from mock, NNA-exposed or recovered XPA depleted BEAS-2B cells. Data represent the mean of ± SEM for N = 3. NNA induced damage was almost completely repaired as indicated by the regain of fluorescence only in BEAS-2B cells (6B, compare lane 1 and 3), however, the damage was only slightly repaired in TC-NER deficient XPA cells (6D, compare lane 1 and 3).

To provide evidence that transcription-coupled NER (TC-NER) is required for removal of lesions produced by NNA exposure, we measured recovery of RNA synthesis in NER-deficient cells, as compared with normal BEAS-2B cells. For cells with NER deficient background, we down-regulated NER protein XPA by siRNA from BEAS-2B cells. Both XPA down-regulated and normal BEAS-2B cells were exposed to 10 μM NNA for 24 h or mock exposure (Fig 6). After the exposure, cells were prepared for IF as described above. In a parallel experiment, NNA was washed out after 24 h of exposure and cells were allowed to recover for 24 h in DMEM medium containing 10% FBS, followed by EU incorporation and *Click IT* reaction (Invitrogen) according to the manufacturer instruction. Quantification of the fluorescence intensity showed that 10 μM NNA exposure reduced RNA synthesis in both BEAS-2B (Fig 6A & 6B, middle panel and lane 2) and XPA down-regulated (Fig 6C & 6D, middle panel and lane 2) cells. However, RNA synthesis recovered almost to baseline levels after a 24 h repair period (Fig 6A & 6B, panel 3 and lane 3) in repair proficient BEAS-2B cells but only slightly recovered in XPA down-regulated BEAS-2B cells (Fig 6C & 6D, panel 3 and lane 3). These results suggest that intact NER is required for resuming transcription, presumably by removal of NNA-induced lesions.

## Discussion

Tobacco use represents an important source of nitrosamine exposure. TSNAs include a wide range of tobacco-related products and commonly form during the tobacco curing process [25,26]. Recently there is a renewed interest in TSNAs as investigators have found the formation of TSNAs from the *in situ* reaction of nicotine absorbed to indoor surfaces with nitrous acid (HONO) in both field and laboratory studies [1,2,7,8,27]. Nicotine, as the most abundant organic compound in smoking with an average of about 8 mg per cigarette and also the main component in THS, has been widely detected on indoor surfaces and smoker’s vehicles, clothe and skin after smoking has stopped [1,2]. It persists for days, weeks and even months [1,2]. HONO is present as a gas in both indoor and outdoor air but it is often at higher levels in indoor environments than outdoors. The main indoor sources of HONO are direct emissions by combustion sources such as unvented gas appliances, smoking, and indirect conversion of NO_2_ and NO on water-containing surfaces in the home [2,28].

Sleiman et al. [2] described the levels of NNA, together with NNK and NNN, formed on indoor surfaces, vehicle, and skin based on calculations of nicotine conversion to NNA (nicotine concentrations were based on previous field studies) [29–31]. It was estimated that substantial amount (3 to 3500 ng per m^2^) of NNA can be present depending on the indoor matrix, vehicle dashboard, skin or clothing [2]. Dermal contact with surface contaminated TSNAs as well as inhalation and ingestion of the TSNA-loaded dust are likely the main exposure route [2]. The concentration range of NNA (0.1 to 10 μM) used in our cell culture-based experiments is considered to be comparable to the real-life exposure levels. Although NNK, NNN and NNA are all detected in THS samples, their levels are or could be lower compared to some of the other nicotine transformation products [1]. However, the genotoxic potential of a chemical compound is not always proportional to its quantity. For instance, NNK is a potent carcinogen with cancer potency of 49 kg mg^-1^d^-1^ [32]. In addition, as mentioned before, a mouse study demonstrated that NNA could be adsorbed through skin [6]. In any case, such studies provided a rationale for further investigation on the potential of NNA to cause adverse biological and health effects.

As mentioned, NNA carcinogenicity is virtually unknown, but a previous mutagenicity assay suggested that NNA is mutagenic but less than NNK in cells expressing P450 CYP2D6 cDNA [11]. In 2013, to investigate the genotoxicity of NNA as it was discovered to be one of the major TSNAs in THS pollution, we employed an alkaline Comet assay to investigate the strand breaks in cultured human cells upon NNA exposure [7]. This method detects a mixture of damages including SSBs, DSBs and DNA cross-links. SSBs are readily repaired but DSBs are lethal. It was reported that a single unrepaired DSB is sufficient to kill a cell or impaired its genomic integrity [33]. In this work, we found that NNA exposure at the environmental relevant concentrations induced significant levels of genotoxic DSBs in exposed human cells with a 53BP1 immunofluorescence assay, suggesting a risk for mutation and cell death.

Another important class of DNA lesions, the oxidative stress-induced base lesions, also plays a key role in environmental mutagenesis and carcinogenesis. Reactive oxygen species (ROS) was detected in THS that causes the accumulation of oxidized base damage in various mammalian cells [7,8]. NNA was also shown to produce 8-oxo-7, 8-dihydro-2′-deoxyguanosine (8-oxodG) in DNA, a major oxidative lesion with potent mutagenic activity (1,21). Some ROS, such as the hydroxyl radical (^•^OH), is highly reactive and capable of oxidizing molecules upon contact. Deoxyguanosine (dG) is particularly vulnerable to oxidation due to its low oxidation potential. The primary oxidation product of dG is 8-oxodG which is generated by the introduction of an oxo group on the C8 position, and addition of a hydrogen atom on the N7 of the imidazole ring of dG. It is expected that NNA-induced oxidized bases are diverse and an important prerequisite for NNA-mediated adverse effects such as the increased mutagenicity observed in NNA-exposed cells [8]. We found increased accumulation of oxidized DNA lesions in multiple human genes as well as the noncoding region of the genome following exposure of lung cells to NNA by LA-QPCR assay (Figs 1 & S1). Induction of cellular HIF-1α after NNA exposure as shown in this study, which is a stress response protein known to be induced by peroxide, also suggests an increased ROS production or a damage accumulation mediated by ROS.

The results from this work using lung BEAS-2B cells for 53BP1foci formation and detection are consistent with those obtained from the HepG2 cells used in Comet assay as reported before [7]. Induction of DSBs is consistent with increased activation of ATM, phosphorylation of p53 and increased expression of p21 following exposure of BEAS-2B cells to NNA as compared to mock exposure control (Fig 2C). Also, extensive hyper-phosphorylation of RPA32(S4/S8) following exposure of lung cells to NNA (Fig 3C) is consistent with the induction of replication stress as has been found upon THS exposure [13]. Induction of DSBs and replication stress correlate with a significant NNA-induced increase in MN formation. Taken together, although several known THS constituents such as polycyclic aromatic hydrocarbons (PAHs) may contribute to its overall genotoxic potency, NNA in THS might also contribute to such genotoxic effects.

Bulky DNA lesions, generated as a consequence of UV radiation or chemical exposure, are strong blocks to transcribing RNAPII and cause transcriptional stress resulting reduced RNA synthesis. These lesions are repaired by the NER pathway with those in transcribed strands being preferentially repaired by TC-NER at a much faster rate than the global genome repair [34–36]. Dose dependent reduction of RNA synthesis as measured by EU incorporation following exposure of lung cells to NNA is consistent with the induction of bulky DNA lesions. Reduction of RNA synthesis at 10 μM NNA concentration is comparable with the strong UV dose (20 J/m2), suggesting the generation of increased blocking lesions in the transcribed strand.

We also investigated the recovery of RNA synthesis in BEAS-2B cells and compared to that in the isogenic XPA down-regulated cells which are deficient in removing bulky DNA lesions by TC-NER (or global NER). The ability of normal BEAS-2B cells to recover RNA synthesis was cloase to the baseline level but only slight recovery in XPA down-regulated cells was observed after exposure to 10 μM NNA, implying that TC-NER is required for removing NNA exposure-induced lesions, particularly the bulky adducts, in order to resume transcription. The slight recovery in XPA-depleted cells following NNA exposure may suggest the repair of NER independent lesions such as oxidative base damage, or that RNAPIIo can bypass those lesions during transcription [37].

In conclusion, using doses relevant to real-life exposure, we demonstrate that NNA exposure induced the accumulation of DNA damage including DSBs, oxidized base lesions and MN formation in BEAS-2B cells, all of which may lead to genomic instability. The observed replication and transcriptional stress following exposure to NNA may account for its adverse cellular impact and disease mechanism as well. The above effects also suggest that NNA in THS may be a contributing factor in the biological and health impact imposed by exposure to THS.

## Supporting information

S1 Fig NNA exposure induced oxidative DNA damage. BEAS-2B cells were exposed to 0.1, 1 and 10 μM of NNA for 24 h followed by processing of the genomic DNA with Fpg and EndoIII as described in the Methods. Mock exposure (DMEM only) was performed in parallel. (A) Amplification of the long (10.4 kb) and short (250 bp) amplicon of *HPRT* gene. Amplification of the large fragment was normalized to the corresponding short fragment. The bar graph represents the normalized data as relative band intensity with mock-exposed sample arbitrarily set to 100.
(TIF)

S2 Fig Induction of HIF-1 α following exposure of BEAS-2B cells to NNA. BEAS-2B cells were exposed to 1 and 10 μM NNA for 24h followed by Western analysis with HIF-1 α antibody. XPB was used as loading control. Quantification shown alongside.
(TIF)

S3 Fig Knock-down of XPA from BEAS-2B cells by siRNA. BEAS-2B cells were transfected with control (siCTRL) or XPA (siXPA) siRNA as described in Methods. Seventy two hours after transfection extracts were made and were tested for XPA knock-down by Western. XPB was used as loading control.
(TIF)

S1 Raw images (PDF)

## Abbreviations

DSB: Double-strand break
SSB: Single-strand break
TSNAs: Tobacco-specific nitrosamines
NNA: 4-(Methylnitrosamino)-4-(3-pyridyl) butanal
NNK: 4-(methylnitrosamino)-1-(3-pyridyl)-1-butanone
NNN: N’-nitrosonornicotine
MSS: Mainstream smoke
SHS: Secondhand smoke
THS: Thirdhand smoke

## References

[1] Jacob III P, Benowitz NL, Destaillants H, Gundel L, Hang B, Martin-Green M, et al (2017) Thirdhand Smoke: New evidence, challenges and future directions. Chem Res Toxicol 17; 30 [1] 270–294. doi: 10.1021/acs.chemrestox.6b00343 28001376 PMC5501723

[2] Sleiman M, Gundel LA, Pankow JF, Jacob P 3rd, Singer BC, Destaillats H (2010) Formation of carcinogens indoors by surface-mediated reactions of nicotine with nitrous acid, leading to potential thirdhand smoke hazards. Proc Natl Acad Sci USA. Apr 13;107[15]: 6576–81.doi: 10.1073/pnas.0912820107 20142504 PMC2872399

[3] Bahl V, Shim HJ, Jacob P 3rd, Dias K, Schick SF, Talbot P (2016) Thirdhand smoke: Chemical dynamics, cytotoxicity, and genotoxicity in outdoor and indoor environments. Toxicol In Vitro. Apr; 32:220–31. doi: 10.1016/j.tiv.2015.12.007 26689327 PMC5526588

[4] Whitlatch A, Schick S (2019) Thirdhand Smoke at Philip Morris. Nicotine Tob Res. Nov 19;21[12]:1680–1688. doi: 10.1093/ntr/nty153 30053240

[5] Hang B (2010) Formation and repair of tobacco carcinogen-derived bulky DNA adducts. J. Nuclei Acids. Dec 20; 2010:709521. doi: 10.4061/2010/709521 21234336 PMC3017938

[6] Jacob, P. III, Havel, C., Yu, L., Adhami, N., Flores, C., Martins- Green, M., et al (2014) Determination of Two Metabolites of the Tobacco-Specific Nitrosamine NNA [4-[methylnitrosamino]-4-[3- pyridyl]butanal] in Mouse Urine. Application to a Dermal Absorption Study in Mice, Presented at the 20th Annual Meeting of the Society for Research on Nicotine and Tobacco [SRNT], February 5−8, Seattle WA, USA. Society For Research on Nicotine and Tobacco, Madison, WI, USA.

[7] Hang B, Sarker AH, Havel C, Saha S, Hazra TK, Schick S, et al (2013) Thirdhand smoke causes DNA damage in human cells. Mutagenesis, 28, 381–9. doi: 10.1093/mutage/get013 23462851 PMC3681537

[8] Hang B, Wang Y, Huang Y, Wang P, Langley SA, Bi L, et al (2018) Short-term early exposure to thirdhand cigarette smoke increases lung cancer incidence in mice. Clin Sci [Lond]., Feb 28; 132[4]:475488. doi: 10.1042/CS20171521 . 29440622 PMC6365648

[9] Sarker AH, Trego KS, Zhang WG, Jacob III P, Snijders A, Mao JH, et al (2020) Thirdhand Smoke Exposure Causes Replication Stress and Impaired Transcription in Human Lung Cells. Environ Mol Mutagen., Apr 8. doi: 10.1002/em.22372 . 32267018 PMC7363442

[10] Hang B, Wang P, Zhao Y, Sarker A, Chenna A, Xia Y, et al (2017) Adverse Health Effects of Thirdhand Smoke: From Cell to Animal Models. Int J Mol Sci. Apr 28;18[5]. pii: E932. doi: 10.3390/ijms18050932 28452951 PMC5454845

[11] Crespi CL, Penman BW, Gelboin HV, Gonzalez FJ (2019) A tobacco smoke-derived nitrosamine, 4-[methylnitrosamino]-1-[3-pyridyl]-1-butanone, is activated by multiple human cytochrome P450s including the polymorphic human cytochrome P4502D6. Carcinogenesis. Jul;12[7]:1197–201. doi: 10.1093/carcin/12.7.1197 . 2070484

[12] Hecht SS., Chen CB, Hirota N, Ornaf RM, Tso T C and Hoffmann D (1978) Tobacco specific nitrosamines: formation from nicotine in vitro and during tobacco curing and carcinogenicity in strain A mice. J. Natl. Cancer Inst.60, 819–824.] doi: 10.1093/jnci/60.4.819 633391

[13] Sarker AH, Chatterjee A, Willams M, Lin S, Havel C, Jacob P, et al (2014) NEIL2 Protects against oxidative DNA damage induced by sidestream smoke in human cells. PLoS ONE 9[3]; e90261. doi: 10.1371/journal.pone.0090261 24595271 PMC3945017

[14] Santos JH, Meyer JN, Mandavilli BS, Van Houten B (2006) Quantitative PCR-based measurement of nuclear and mitochondrial DNA damage and repair in mammalian cells. Methods Mol Biol., 314:183–99. doi: 10.1385/1-59259-973-7:183 16673882

[15] Nakazawa Y, Yamashita S, Lehmann AR and Ogi T (2010) A semi-automated non-radioactive system for measuring recovery of RNA synthesis and unscheduled DNA synthesis using ethynyluracil derivatives. DNA Repair, 9 [2010] 506–516. doi: 10.1016/j.dnarep.2010.01.015 20171149

[16] Fenech M, Chang WP, Kirsch-Volders M, Holland N, Bonassi S, Zeiger E (2003) *HUMN* project; detailed description of the scoring criteria for the cytokinesis-block micronucleus assay using isolated human lymphocyte culture. Mutat Res., Jan 10; 534[1–2]: 65–75. doi: 10.1016/s1383-5718(02)00249-8 . 12504755

[17] Rydberg B, Chun E, Groesser T (2007) Relative Biological effectiveness of high-energy iron ions for micronucleus formation at low doses. Radiation Research, 168[6]; 675–682. doi: 10.1667/RR0967.1 18088180

[18] Trego KS, Groesser T, Davalos AR, Parplys AC, Zhao W, Nelson MR, et al (2016) Non-catalytic Roles for XPG with BRCA1 and BRCA2 in Homologous Recombination and Genome Stability. Mol Cell, Feb 18;61[4]:535–546. doi: 10.1016/j.molcel.2015.12.026 Epub 2016 Jan 28. . 26833090 PMC4761302

[19] Jung SN, Yang WK, Kim J, Kim HS, Kim EJ, Yun H, et al (2008) Reactive oxygen species stabilize hypoxia-inducible factor-1 alpha protein and stimulate transcriptional activity via AMP-activated protein kinase in DU145 human prostate cancer cells. Carcinogenesis. Apr;29[4]:713–21. doi: 10.1093/carcin/bgn032 Epub 2008 Feb 6. 18258605

[20] Lavin MF and Kozlov S (2007) ATM activation and DNA damage. Cell cycle, Apr 15; 6[8]: 931–42. doi: 10.4161/cc.6.8.4180 17457059

[21] Hang B, Iavarone A, Havel C, et al. (2014) NNA, a thirdhand smoke constituent, induces DNA damage in vitro and in human cells. 247th National Meeting of the American Chemical Society [ACS] with press release; March 16–20. Dallas, TX.

[22] Lavin MF and Kozlov S (2007) ATM activation and DNA damage. Cell cycle, Apr 15; 6[8]: 931–42. doi: 10.4161/cc.6.8.4180 17457059

[23] Oakley GG and Patrick SM (2012) Replication protein A: directing traffic at the intersection of replication and repair. Front Biosci., 15: 883–900. 10.2741/3652 PMC3399740 20515732

[24] Scharer OD (2013) Nucleotide Excision Repair in Eukaryotes. Cold Spring Harb Perspect Biol., 2013;5: a012609. doi: 10.1101/cshperspect.a012609 24086042 PMC3783044

[25] Brown BG, Borschke AJ, and Doolittle DJ (2003) An Analysis of the Role of Tobacco-Specific Nitrosamines in the Carcinogenicity of Tobacco Smoke. Nonlinearity Biol Toxicol Med. Apr; 1[2]: 179–198. doi: 10.1080/15401420391434324 19330121 PMC2651603

[26] Andersen RA, Fleming PD, Burton HR, Hamilton-Kemp TR, Sutton TG (1989) *N*′-Acyl and *N*′-nitroso pyridine alkaloids in alkaloid lines of Burley tobacco during growth and air curing. J Agric Food Chem. 37[1]:44–50.

[27] Dhall S, Alamat R, Castro A, Sarker AH, Mao JH, Chan A, et al (2016) Tobacco toxins deposited on surfaces [third hand smoke] impair wound healing. Clin Sci [Lond]. Jul 1;130 [14]:1269–84. doi: 10.1042/CS20160236 27129193

[28] Spicer CW, et al. (1993) The prevalence of nitrous acid in indoor air and its impact on NO2measurements made by passive samplers; Proceedings of the 6th International Conference of Indoor Air Quality and Climate; Helsinki, Finland. 1993; Helsinki: Finnish Society of Indoor Air Quality and Climate; 1993. pp. 277–282.

[29] Matt GE, et al. (2004) Households contaminated by environmental tobacco smoke: Sources of infant exposures. Tob Control 13:29–37. doi: 10.1136/tc.2003.003889 14985592 PMC1747815

[30] Weschler CJ and Nazaroff WW (2008) Semivolatile organic compounds in indoor environments. Atmos Environ 42:9018–9040.

[31] Destaillats H, Singer BC, Lee SK and Gundel LA (2006) The effect of ozone on nicotine desorption from model surfaces: Evidence for heterogeneous chemistry. Environ Sci Technol 40:1799–1805. doi: 10.1021/es050914r 16570600

[32] Pankow JF, Watanabe KH, Toccalino PL, Luo W, Austin DF(2007) Calculated cancer risks for conventional and “potentially reduced exposure product” cigarettes. Cancer Epidem Biomar 2007; 16:584–592. doi: 10.1158/1055-9965.EPI-06-0762 17372256

[33] Jackson SP (2002) Sensing and repairing DNA double-strand breaks. Carcinogenesis, V 23,5; 687–696. doi: 10.1093/carcin/23.5.687 12016139

[34] Lans H, Hoeijmakers JH, Vermeulen W and Marteijin JA (2019) The DNA damage response to transcription stress. Nat Rev Mol Cell Biol, Dec; 20[12];766–784. doi: 10.1038/s41580-019-0169-4 31558824

[35] Hanawalt PC and Spivak G (2008) Transcription-coupled DNA repair: two decades of progress and surprises. Nat Rev Mol Cell Biol, Dec;9[12]:958–70. doi: 10.1038/nrm2549 19023283

[36] Gregersen LH and Svejstrup JQ (2018) The cellular response to transcription-blocking DNA damage. Trends in Biochemical Sciences, May; 43[5]; 327–341. doi: 10.1016/j.tibs.2018.02.010 . 29699641 PMC5929563

[37] Saxowsky TT, Meadows KL, Klungland A, Doetsch PW (2008) 8-oxoguanine-mediated Transcriptional Mutagenesis causes Ras Activation in Mammalian Cells. Proc Natl Acad Sci USA 105[48]:18877–18882. doi: 10.1073/pnas.0806464105 19020090 PMC2596238

