# Supplementary figures and images for: Tobacco-specific nitrosamine 1-(N-methyl-N-nitrosamino)-1-(3-pyridinyl)-4-butanal (NNA) causes DNA damage and impaired replication/transcription in human lung cells
Source: PLoS One. 2022 May 16;17(5):e0267839. doi: 10.1371/journal.pone.0267839 (PMC9109921; doi:10.1371/journal.pone.0267839)

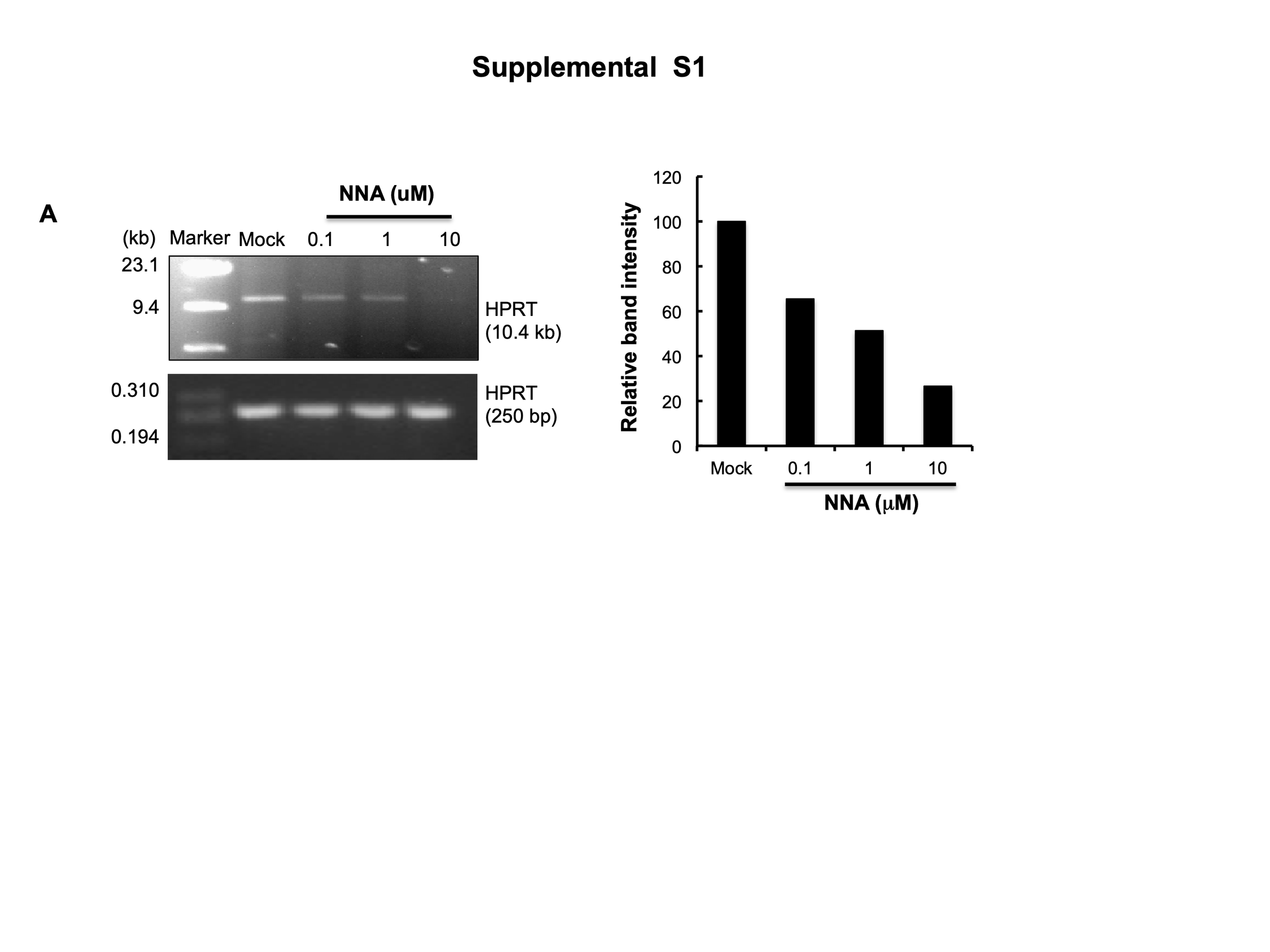

Supplement: S1 Fig — BEAS-2B cells were exposed to 0.1, 1 and 10 μM of NNA for 24 h followed by processing of the genomic DNA with Fpg and EndoIII as described in the Methods. Mock exposure (DMEM only) was performed in parallel. (A) Amplification of the long (10.4 kb) and short (250 bp) amplicon of HPRT gene. Amplification of the large fragment was normalized to the corresponding short fragment. The bar graph represents the normalized data as relative band intensity with mock-exposed sample arbitrarily set to 100. (TIF) [file pone.0267839.s001.tif]

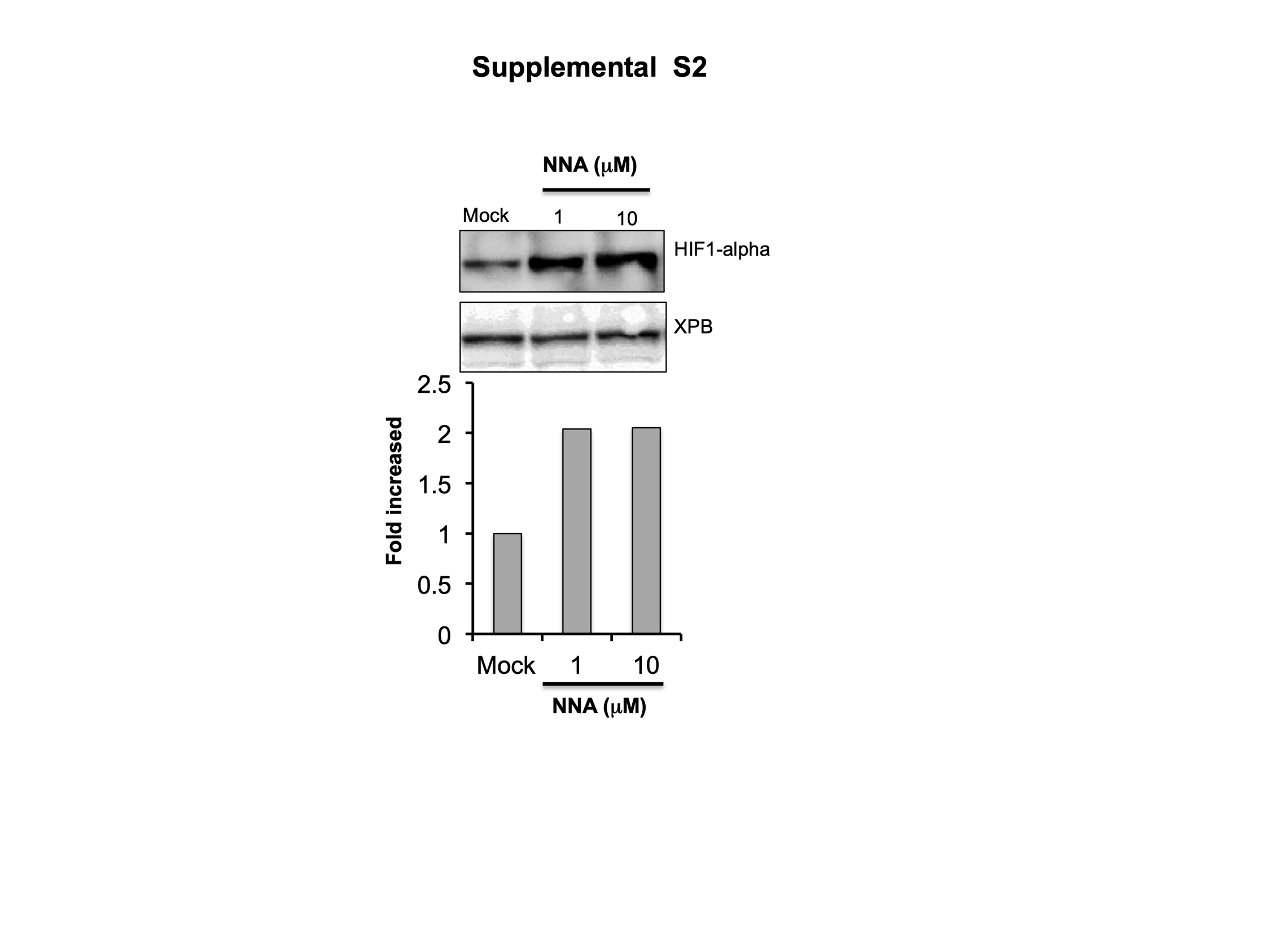

Supplement: S2 Fig — BEAS-2B cells were exposed to 1 and 10 μM NNA for 24h followed by Western analysis with HIF-1 α antibody. XPB was used as loading control. Quantification shown alongside. (TIF) [file pone.0267839.s002.tif]

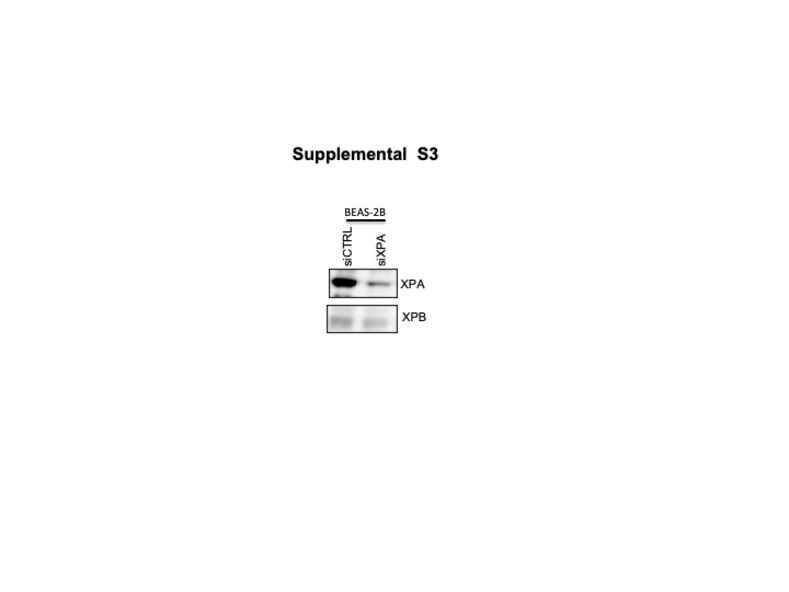

Supplement: S3 Fig — BEAS-2B cells were transfected with control (siCTRL) or XPA (siXPA) siRNA as described in Methods. Seventy two hours after transfection extracts were made and were tested for XPA knock-down by Western. XPB was used as loading control. (TIF) [file pone.0267839.s003.tif]
