# Supplementary material for: Tobacco-specific nitrosamine 1-(N-methyl-N-nitrosamino)-1-(3-pyridinyl)-4-butanal (NNA) causes DNA damage and impaired replication/transcription in human lung cells
Source: PLoS One. 2022 May 16;17(5):e0267839. doi: 10.1371/journal.pone.0267839 (PMC9109921; doi:10.1371/journal.pone.0267839)
Supplement: S1 Raw images — (PDF) [file pone.0267839.s004.pdf]

Figure 1A. 9.4 kb fragment (long)

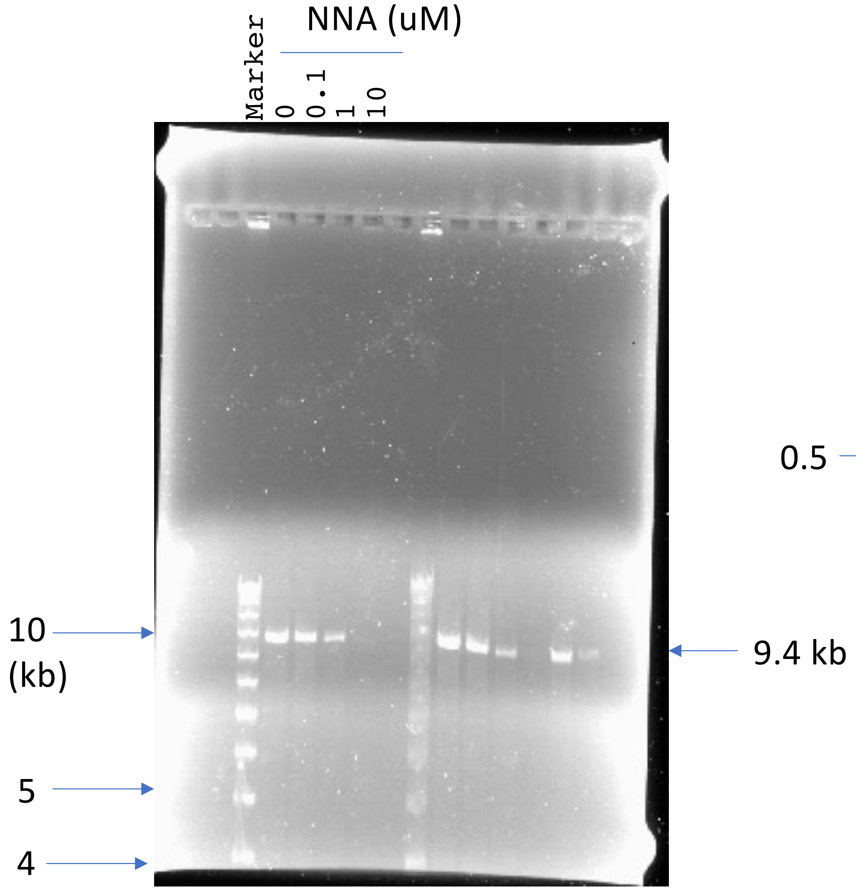

Figure 1A. 192 bp fragment (short)

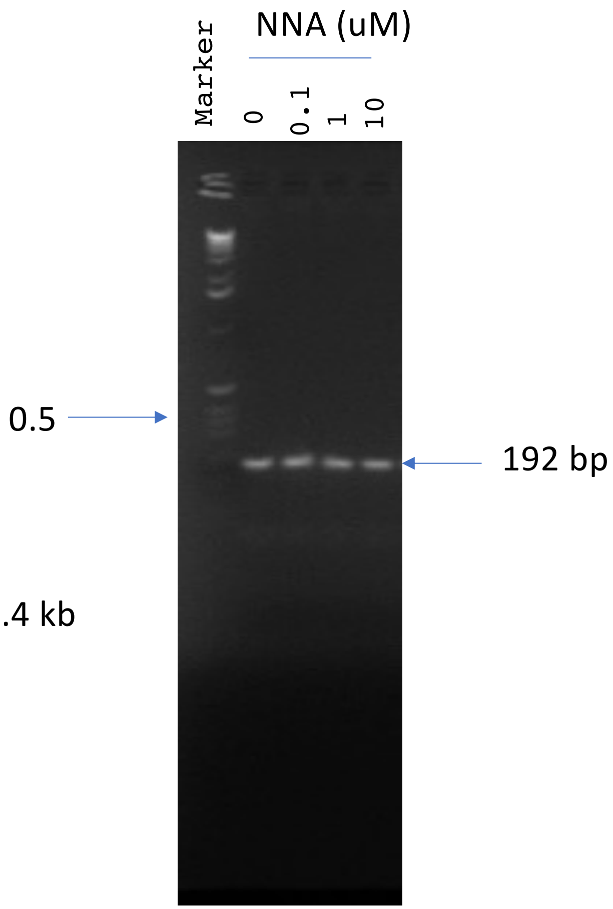

Figure 1C. 9.6 kb fragment

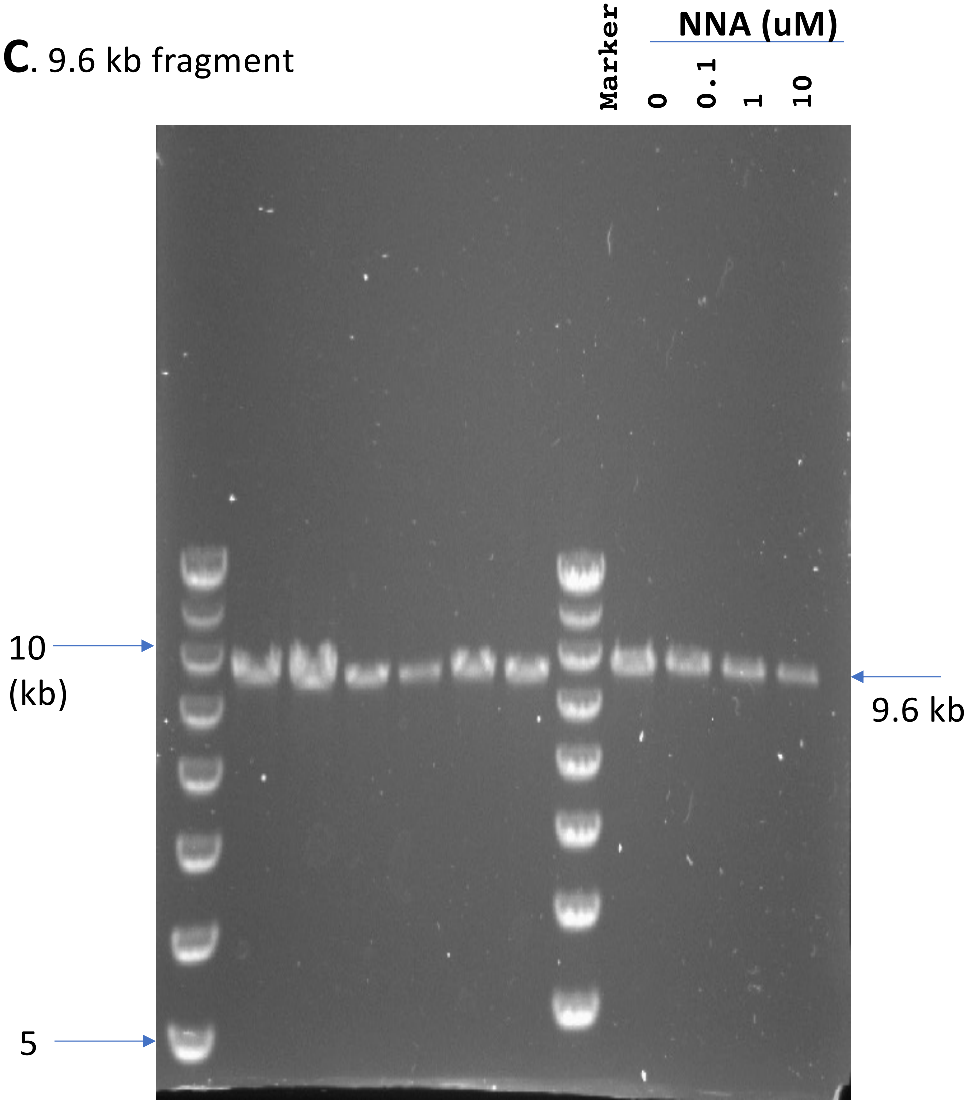

Figure 1C. 294 bp fragment

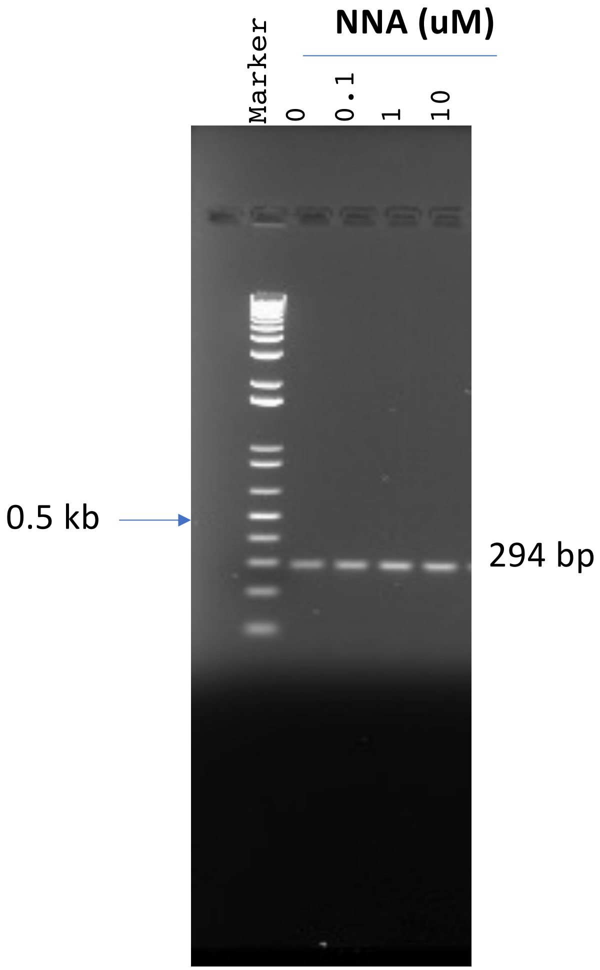

Figure 2C.

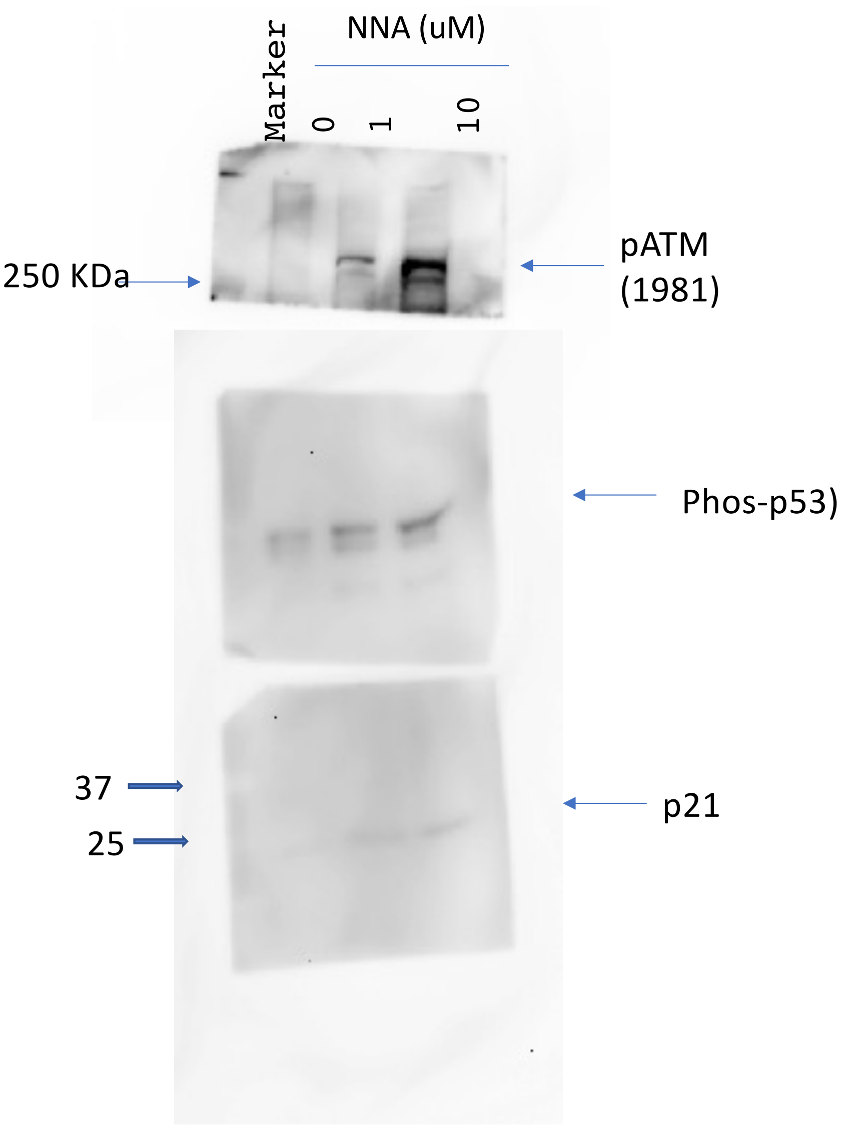
NNA (uM)
Marker
0
1
10
250 KDa
pATM (1981)
Phos-p53)
37
25
p21

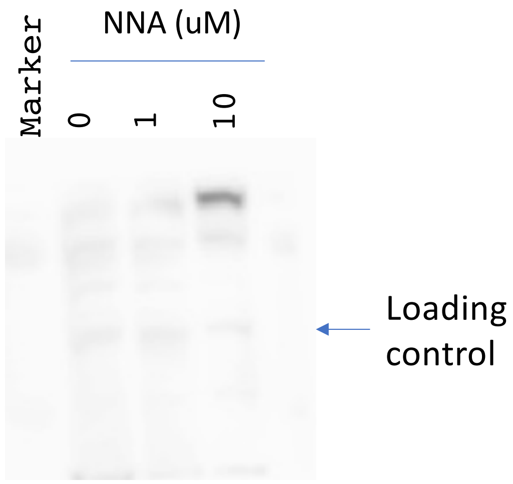
NNA (uM)
Marker
0
1
10
Loading control

Fig. 3C

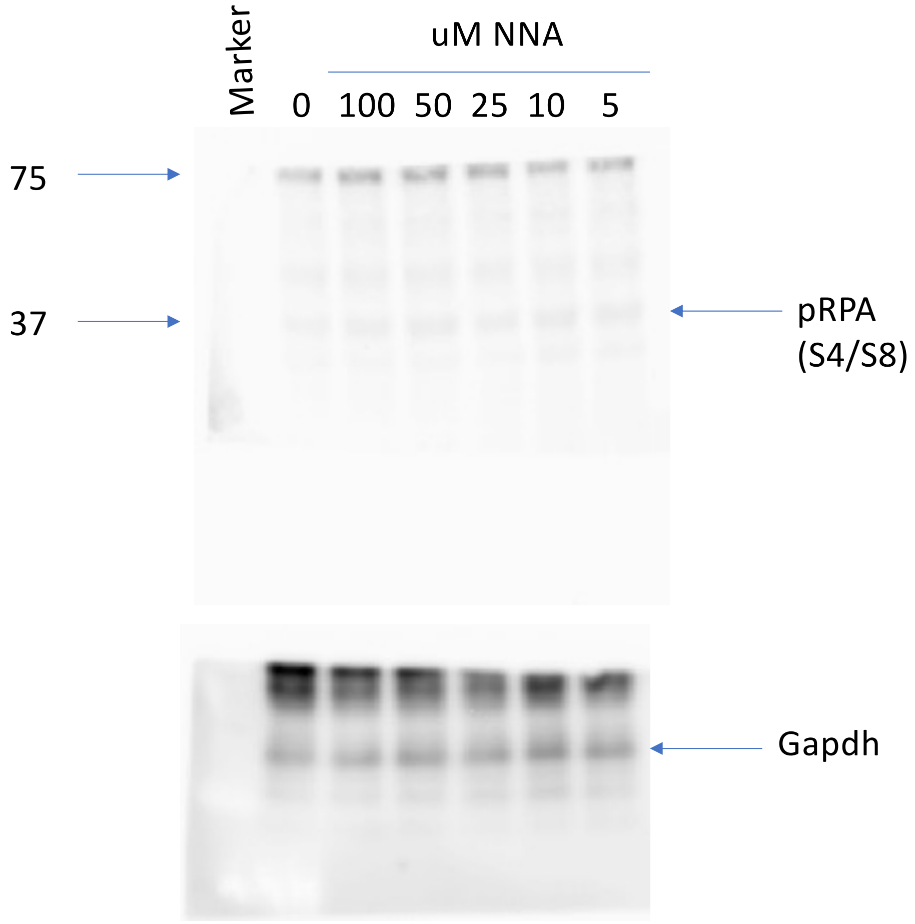

Fig. S1

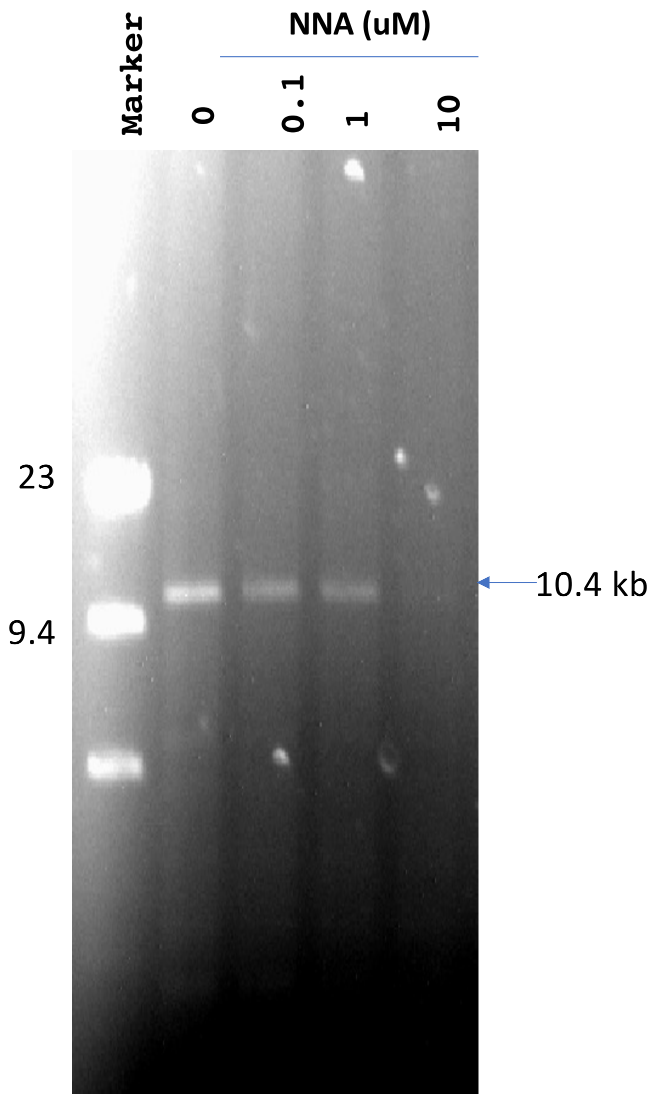
NNA (uM)
Marker
0
0.1
1
10
23
9.4
10.4 kb

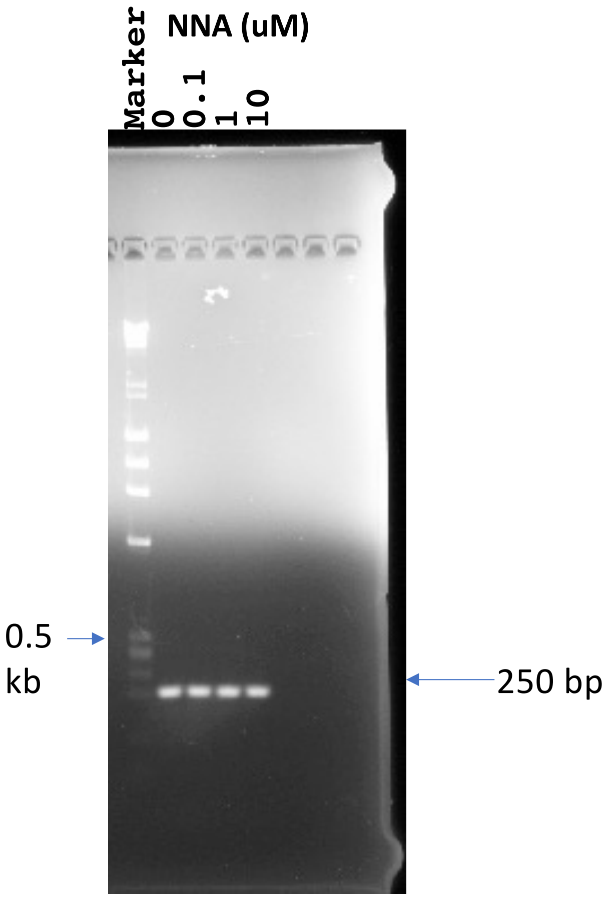
Marker
NNA (uM)
0
0.1
1
10
0.5
kb
250 bp

Fig. S2

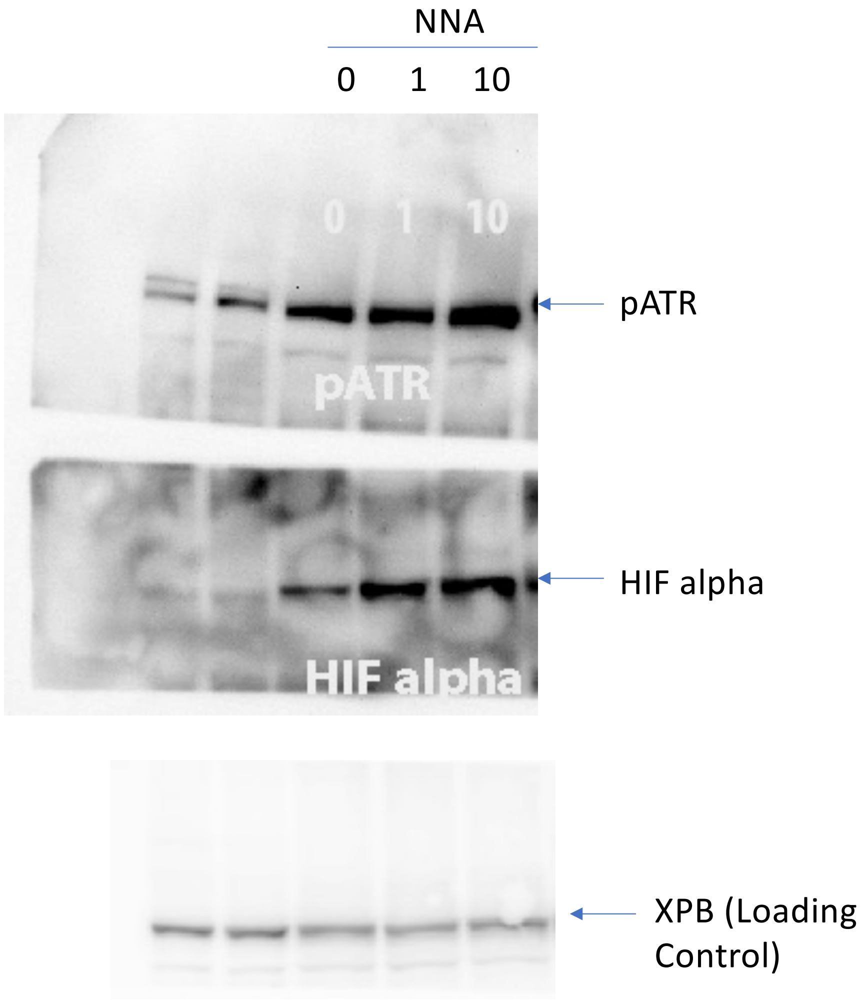
NNA
0
1
10
0 1 10
pATR
pATR
HIF alpha
HIF alpha
XPB (Loading Control)

Fig. S3

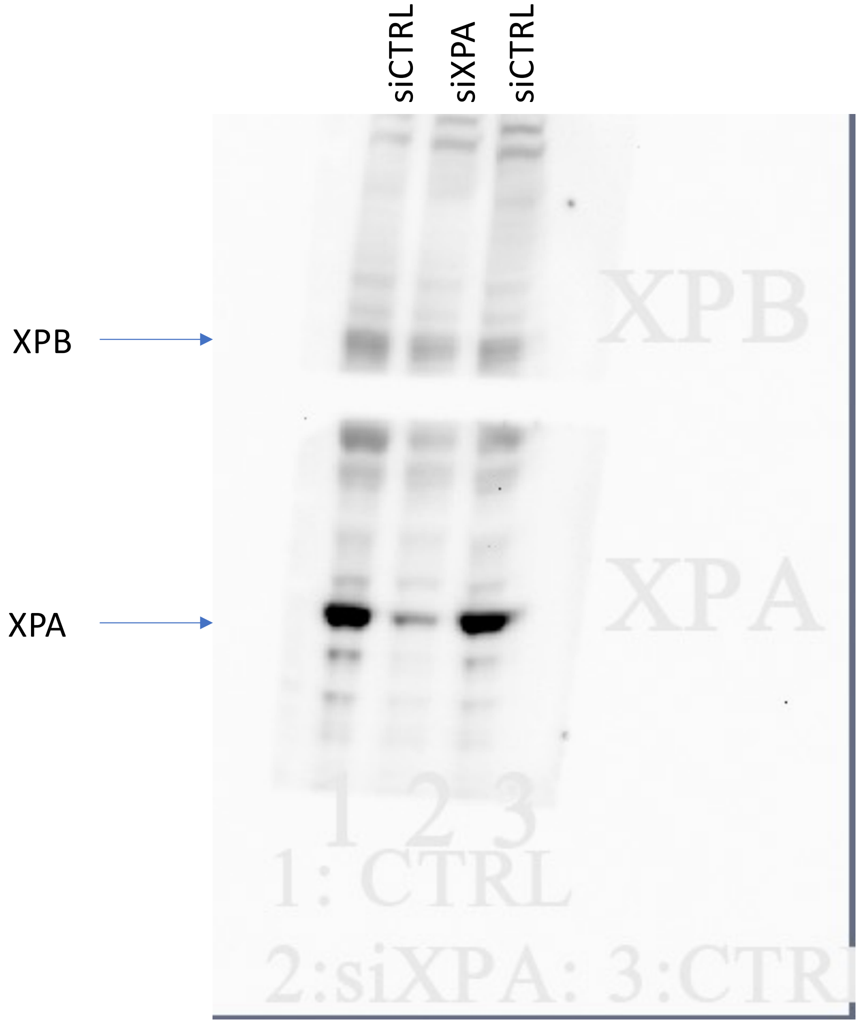

Lane 1# siControl
Lane 2# siXPA
Lane 3# siControl
